# Arrhythmogenic effects of mutated L-type Ca^2+^-channels on an optogenetically paced muscular pump in *Caenorhabditis elegans*

**DOI:** 10.1038/srep14427

**Published:** 2015-09-24

**Authors:** Christina Schüler, Elisabeth Fischer, Lior Shaltiel, Wagner Steuer Costa, Alexander Gottschalk

**Affiliations:** 1 Buchmann Institute for Molecular Life Sciences, Goethe University, Max von Laue Strasse 15, D-60438 Frankfurt, Germany; 2 Institute of Biochemistry, Goethe University, Max von Laue Strasse 9, D-60438 Frankfurt, Germany; 3 Cluster of Excellence Frankfurt—Macromolecular Complexes, Goethe University, Max von Laue Strasse 15, D-60438 Frankfurt, Germany

## Abstract

Cardiac arrhythmias are often associated with mutations in ion channels or other proteins. To enable drug development for distinct arrhythmias, model systems are required that allow implementing patient-specific mutations. We assessed a muscular pump in *Caenorhabditis elegans*. The pharynx utilizes homologues of most of the ion channels, pumps and transporters defining human cardiac physiology. To yield precise rhythmicity, we optically paced the pharynx using channelrhodopsin-2. We assessed pharynx pumping by extracellular recordings (electropharyngeograms—EPGs), and by a novel video-microscopy based method we developed, which allows analyzing multiple animals simultaneously. Mutations in the L-type VGCC (voltage-gated Ca^2+^-channel) EGL-19 caused prolonged pump duration, as found for analogous mutations in the Ca_v_1.2 channel, associated with long QT syndrome. *egl-19* mutations affected ability to pump at high frequency and induced arrhythmicity. The pharyngeal neurons did not influence these effects. We tested whether drugs could ameliorate arrhythmia in the optogenetically paced pharynx. The dihydropyridine analog Nemadipine A prolonged pump duration in wild type, and reduced or prolonged pump duration of distinct *egl-19* alleles, thus indicating allele-specific effects. In sum, our model may allow screening of drug candidates affecting specific VGCCs mutations, and permit to better understand the effects of distinct mutations on a macroscopic level.

The heart beat results from a highly regulated series of physiological events. Cardiac depolarization triggers voltage gated Na^+ ^-channels and VGCCs that shape the rise and plateau phases (T-type VGCCs and L-type VGCCs, respectively) of the cardiac action potential (AP)^1^,^2^. The AP is followed by repolarization, involving several types of K^ + ^-channels, and a resting phase^3^. The period between ventricular depolarization and repolarization defines the QT interval in the electrocardiogram^4^. The rise in cytosolic Ca^2+^ triggers ryanodine receptor 2 (RyR2), a Ca^2+^-activated Ca^2+^ release channel in the sarcoplasmic reticulum (SR), thus evoking contraction. Following repolarization, Ca^2+^ is extruded from the cytosol, by SR membrane Ca^2+^-pumps and the Na^+^/Ca^2+^-exchanger (NCX) in the plasma membrane, jointly leading to muscular relaxation^5^.

Cardiac arrhythmias constitute prevalent forms of heart disease. Arrhythmias can affect heart rate, i.e. faster or slower than normal (tachycardia or bradycardia, with >100 or <60 beats per minute, respectively), or they disturb the regular rhythm and sequence of physiological events constituting a heartbeat, for example in aged hearts^6^, or following damage (e.g. artery disease, heart attack)^7^. Last, arrhythmias are caused by mutations in genes relevant for generation, maintenance and termination of the cardiac AP, as well as intracellular Ca^2+^ handling^8^. For example, Long QT (LQT) syndromes involve a number of genes affecting the duration of the QT interval (prolonging it), like K^+^ channels, and also the L-type VGCC, Cav1.2 (CACNA1C). Mutations in Cav1.2 lead to Timothy syndrome (LQT8), and usually delay inactivation of L-type channels, thus extending the AP plateau phase^9^,^10^,^11^.

Drugs that accelerate inactivation of L-type VGCCs, like 1,4-dihydropyridines (DHPs), are effective in LQT8 patients. However, DHPs broadly affect L-type channels, thus causing side effects. As more specific mutations in Cav1.2 are being identified in LQT8 patients (and for other genes affecting other arrhythmias; see e.g. a database at http://triad.fsm.it/cardmoc/), a demand for new effective drugs, even acting specifically on distinct mutations is generated^12^,^13^. Therefore, genetically amenable systems allowing for straightforward drug screening, in a mutation-specific manner, would be beneficial. Patient-specific cardiomyocytes derived from induced pluripotent stem cells can be used to assess aspects of arrhythmia on a cellular basis, but are too expensive for screening purposes and cannot recapitulate cardiac arrhythmias in the context of the whole heart. A LQT model based on mutations in the KCNH2 channel was previously generated in zebrafish^14^; yet, introducing mutations and transgenes is slow. The nematode *Caenorhabditis elegans* is a genetically accessible experimental system with a 2.5 day life cycle, enabling straightforward (and cheap) mass cultivation, i.e. properties beneficial for transgenesis and drug screening. The *C. elegans* feeding organ, the pharynx, is a rhythmically active muscular pump^15^, with many properties similar to the vertebrate heart (but also dissimilarities)^16^. It consists of 20 muscle cells (and accessory cells), connected by gap junctions as in the heart, forming an elongated structure with an axial lumen and anterior and terminal bulbs (Fig. 1a, lower panel). The mostly autonomous pharyngeal nervous system is required for (fast) pumping and modulation of pharynx activity in presence or absence of bacterial food^17^,^18^,^19^. The pharynx pumps with up to 4 Hz in the presence of bacteria, which are sucked in and moved through procorpus and anterior bulb, to be collected in the anterior isthmus. Isthmus peristalsis then transports bacteria to the terminal bulb, where they are mechanically lysed^20^. Like in the heart, pharynx contractions continue in the absence of coordinate neuronal input, but require acetylcholine. Ion channels facilitating pharynx action are known and the pharyngeal AP (well resembling the human cardiac AP, as it exhibits a plateau) was thoroughly characterized. Fast pharynx pumping requires EAT-2 nicotinic acetylcholine receptors (nAChRs)^21^, replacing voltage gated sodium channels. T-type and L-type VGCCs (encoded by *cca-1* and *egl-19*, respectively) shape the rise- and plateau phases of the pharyngeal AP^22^. Repolarization is modulated by glutamate-gated chloride channels, and executed by voltage-gated K^+^-channels, encoded by *exp-2* (ref. 23) which, though homology is low, is functionally similar to the mammalian HERG channel, but also UNC-103 K^+^-channels. Pharynx muscle also expresses the UNC-68 ryanodine receptor and the SR Ca^2+^ storage protein CSQ-1, similar to human CASQ2 (refs. 24, 25, 26). Mutations in these genes affect pharynx pumping and its physiological and electrical properties. They may thus serve to establish a model system for homologous mutations found in humans. Some aspects of pharynx cell biology, however, point to convergent development of the two organs, like origin from ectodermal (pharynx) vs. mesodermal tissue (heart)^16^.

Pharynx pumping can be recorded by EPGs, analogous to electrocardiograms. The head of the animal is sucked into a pipette for extracellular recording. Either whole animals, or cut head preparations are used, the latter enabling more accurate measurements of pump rate and duration, and distinguishing muscle activity of different parts of the pharynx, as well as activity of pharyngeal neurons^27^,^28^. Pharynx pumping is influenced by the presence of food, but also by neuromodulators like serotonin^29^,^30^. Drugs can be applied either to intact animals, or to cut heads, where drug access is more direct. Spontaneous pharynx pumping is not as regular as required to assess rhythmicity and the consequences of arrhythmogenic mutations. Thus, experimental means of pacing the pharynx to a precisely regular beat are necessary to generate a *C. elegans* test system for analyzing arrhythmogenic mutations, as well as anti-arrhythmogenic drugs. For high throughput, non-invasive methods for pacing would be beneficial, which may be achieved by optogenetics in the transparent animal, using the light-gated ion channel channelrhodopsin (ChR2)^31^,^32^,^33^. Here, we establish the pharynx as an optogenetically paced system. By ChR2-mediated depolarization, we achieved pharynx pumping up to 6 Hz. Mutations in the L-type VGCC EGL-19 affected pump duration and ability of the pharynx to achieve high pump rates, similar to a LQT syndrome. Video microscopy and kymographic analyses enabled high throughput, while deducing key parameters of the AP, and also revealed arrhythmic episodes during prolonged pacing. Importantly, the DHP analog nemadipine A (from now on, nema-A) permitted reverting the prolonged pump duration of a specific allele of *egl-19*, while it had opposing effects on wild type (wt) and other *egl-19* alleles. Optogenetic pharynx pacing thus facilitates functional analysis of VGCC alleles in the context of a whole organ, and enables straightforward screening for novel anti-arrhythmogenic drugs.

## Results

### ChR2 enables optogenetic pacing of the *C. elegans* pharynx

**Figure 1:**
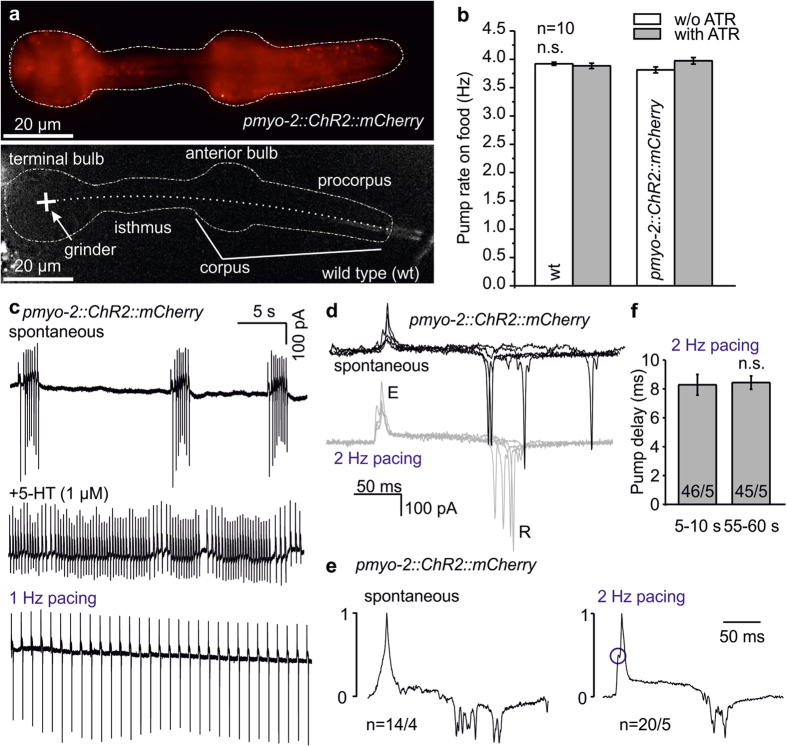
ChR2 expressed in pharyngeal muscle cells (PMCs) enables photostimulation and light-induced pumping with high reproducibility. (**a**) Expression of ChR2(H134R)::mCherry in PMCs, compared to a non-transgenic control. Dashed lines indicate pharynx position, dotted line indicates pharynx lumen, structural features of the pharynx are named. Scale bars: 20 μm. (**b**) The pharynx pump rate on food (visually counted) is unaffected by ChR2 expressed in PMCs (compared to wild type—wt), regardless of cultivation in absence or presence of 10 μM ATR (n = 10 animals each). (**c**) EPG recordings of activity in pharynxes of animals expressing ChR2::mCherry in PMCs. Compared are spontaneous activity (upper trace), activity stimulated by addition of serotonin (5-HT, 1 μM; middle trace), or in response to 1 Hz optical pacing (lower panel). (**d**) Comparison of overlaid original EPG recordings of spontaneous (black) and 2 Hz (10 ms, 470 nm) stimulated (grey) EPGs from four pharynxes expressing ChR2::mCherry. E (excitation/ contraction of pharynx) and R (relaxation/repolarization) peaks are indicated, traces have been aligned to the E spike. (**e**) Averaged, E-peak normalized EPG traces of spontaneous (14 traces) or 2 Hz paced pumps (20 traces) from different pharynxes (n = 4–5). A peak preceding the E-spike is observed in averaged 2 Hz paced pump traces, likely elicited by ChR2-photocurrents in PMCs, indicated by a blue circle. (**f**) Pump delay (time between start of stimulation pulse and occurrence of E-peak) is consistent during long recording periods, and was averaged from the indicated number of pumps/animals, for seconds 5–10 or 55–60 of one minute paced pumping.

We expressed ChR2(H134R)::mCherry (a gain-of-function mutant^31^; from now on, ‘ChR2’ refers to this variant) in pharyngeal muscle cells (PMCs), using the *pmyo-2* promoter. Expression was expected in intracellular and plasma membranes. Diffuse mCherry-fluorescence could be observed in the whole pharynx, whereas no fluorescence was detected in wt animals (Fig. 1a). Spontaneous pump rate of the pharynx, in presence of bacteria, in PMC-ChR2 animals or wt, cultivated with or without the ChR2 chromophore all-*trans* retinal (ATR) was unaltered, indicating that PMCs were not affected by ChR2 expression (Fig. 1b).

We tested whether pharynx pumping could be paced optically. To assess electrical events associated with pharynx pumping, we used EPG recordings. Spontaneous pharynx activity is unsteady (Fig. 1c, upper panel): Periods of fast pumping alternate with periods of low or no pump activity. Addition of serotonin stimulates pumping^30^, leading to ongoing activity, yet the pump rate is too variable to allow detecting ‘arrhythmic’ events (Fig. 1c, middle panel). In contrast, when we applied pacing light pulses (1 Hz, 470 nm, 10 ms, 1.5 mW/mm^2^), ChR2-transgenic pharynxes of animals showed a steady 1 Hz rhythm (Fig. 1c, lower panel). We analyzed the EPGs in detail. Compared to spontaneous pumping, the pump duration, i.e. period between E- (excitation, contraction of terminal bulb) and R (relaxation/repolarization of corpus^27^,^28^) peaks was much more reproducible between animals and between different pumps in pharynxes optically paced at 2 Hz (Fig. 1d), and in averaged, E-peak normalized traces, R-peaks were much less distributed in paced pharynxes (Fig. 1e). Paced pharynxes exhibited an extra peak in the averaged EPG preceding the E-peak, representing ChR2 photocurrents. The delay between the light pulse and the E-peak was very reproducible with low jitter for 2 Hz paced pharynxes, regardless of whether this was determined early (5–10 s) or late (55–60 s) during 1 min, 2 Hz paced pump trains (8.29 ± 0.72 and 8.44 ± 0.46 ms, respectively), showing high reliability (Fig. 1f).

**Figure 2:**
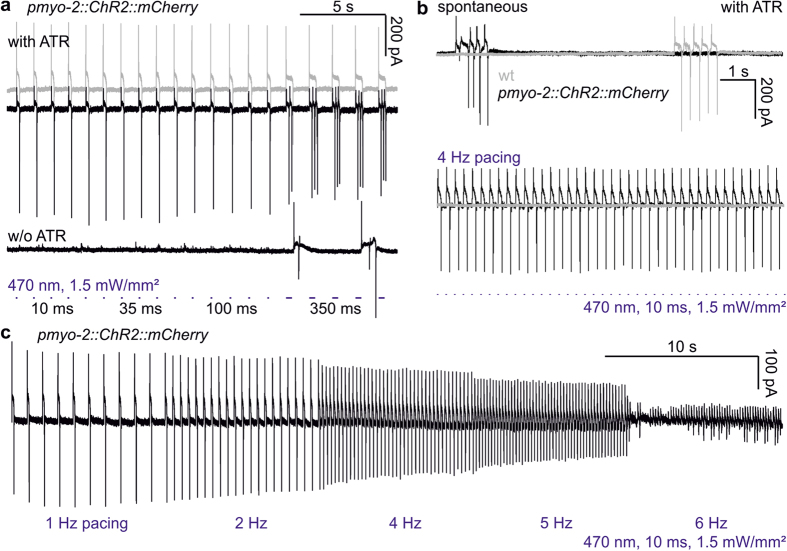
ChR2-mediated stimulation of the pharynx induces highly regular pumping at frequencies up to 7 Hz. (**a**) Pumping in ChR2::mCherry expressing pharynxes, recorded as EPGs, stimulated by light pulses of different length (10, 35, 100, 350 ms), causing accordingly increased pump duration (grey trace), or multiple pumps (top black trace) during 350 ms stimuli. Pharynxes of animals raised without ATR exhibit non-specific pump events (bottom black trace). (**b**) Pumping can be stimulated at 4 Hz, (470 nm, 10 ms, 1.5 mW/mm^2^; lower black EPG trace), but not in non-transgenic animals (lower grey trace). Upper traces: Non-transgenic (grey) or transgenic pharynxes in the presence of ATR (black) exhibit normal bouts of spontaneous pumping. (**c**) Pumping (single EPG trace) stimulated by increasing frequencies of light pulses as in b, as a ‘stress test’ protocol (pacing frequency increased stepwise up to 6 Hz, as indicated below the trace). This pharynx could not lock in to 6 Hz pacing.

Next, we assessed if and how duration of light pulses would affect pump duration or the number of evoked pumps. At 10, 35 or 100 ms, no difference was observed (n = 6; Fig. 2a; Supplementary Fig. S1). However, 350 ms light pulses caused multiple, or long-lasting pumps. Animals cultivated without ATR showed no pumps in response to short light pulses, though 350 ms light pulses sometimes evoked pumping, possibly an indirect consequence of photophobic signaling by LITE-1 and/or GUR-3 UV-/blue light receptors^34^,^35^. We thus used light pulses of 10 or 35 ms in subsequent experiments. 4 Hz pumping could be readily evoked in ChR2-transgenic animals, but not in non-transgenic animals (Fig. 2b). In a ‘stress test’, pacing was increased every 10 s in 1 Hz increments up to 6 Hz (Fig. 2c; Supplementary Video 1). Most PMC-ChR2 pharynxes could achieve 5 Hz maximal frequency. Many pharynxes would also follow 6 Hz reliably, though they sometimes alternated between 6 and 3 Hz pumping. Rarely, pharynxes could be paced up to 7 Hz (10 ms pulses, 1.5 mW/mm^2^). To enable pacing for long periods (up to 65 s), we settled with 1.5–2 mW/mm^2^ light intensity.

### The pharyngeal nervous system does not influence pump duration in the paced pharynx

**Figure 3:**
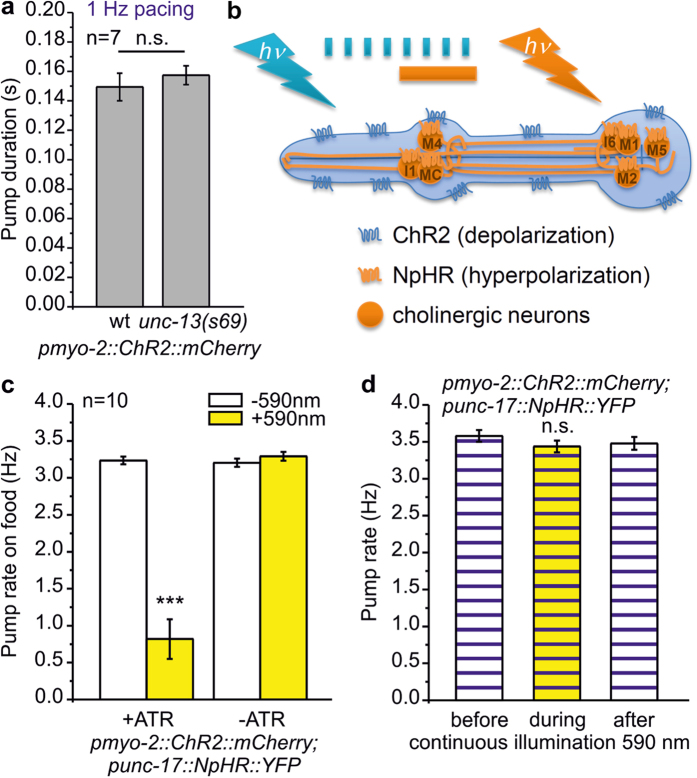
The pharyngeal nervous system does not influence EPG parameters during optogenetically paced pumping. (**a**) Pump duration during 1 Hz pacing compared in wild type (wt) and *unc-13(s69)* mutant (n = 7 pharynxes). (**b**) Schematic depicting transgenic pharynxes used in experiments in (**c**,**d**). The pharynx expresses ChR2 in PMCs and cholinergic pharyngeal neurons express halorhodopsin (NpHR). While ChR2 is activated by 470 nm light pulses (blue tick marks) to induce pumping, cholinergic neurons are hyperpolarized by continuous illumination (1 min) with 590 nm light (yellow bar) (**c**) Spontaneous pump rate in presence of bacteria, in pharynxes as described in B, from animals raised in the absence or presence of ATR, as indicated. Yellow-light induced hyperpolarization of cholinergic neurons affects spontaneous pump rate (t-test with Bonferroni correction, ***P < 0.001). (**d**) Mean pump rate, visually counted for 30 s during 4 Hz optical pacing (35 ms, 470 nm) is not impaired by hyperpolarization of cholinergic neurons using yellow light (middle). Compared is frequency before (left) or after yellow light (right).

The pharynx is innervated by 20 neurons^17^,^18^,^19^. To establish the pharynx as an arrhythmia model, we needed to assess whether neuronal signaling may influence paced pharyngeal pumping. Recent work showed that optogenetic stimulation of pharynx neurons induces pumping^36^. Pharyngeal neurons may also negatively regulate pump rate or duration, possibly counteracting ChR2-mediated depolarization. Pump duration in *unc-13(s69)* mutants, which have abolished chemical synaptic transmission^37^, was no different during 1 Hz pacing (157 ± 6, compared to 149 ± 9 ms in wt; n = 7; Fig. 3a). To look for acute effects of cholinergic neurons on paced pumping in PMC-ChR2 animals, we expressed the hyperpolarizing, light-driven chloride pump halorhodopsin (NpHR)^38^ in cholinergic cells, some of which innervate the pharynx (MC, M1, M2, M4, M5, I1 and I6 neurons; Fig. 3b). We first assessed the effect of neuronal silencing on spontaneous pumping. Photoinhibition of cholinergic neurons (590 nm, 0.5 mW/mm^2^), which led to body relaxation due to silencing of cholinergic neuromuscular junctions^38^ (Supplementary Fig. S2), significantly reduced the pump rate on food (3.24 ± 0.05 vs. 0.82 ± 0.27 Hz; n = 10; Fig. 3c). However, upon optogenetic pacing (3.7 Hz, 35 ms, 470 nm), 1 min photo-inhibition of cholinergic neurons had no significant effect on the pump rate (Fig. 3d). Thus, neuronal malfunction, which could be effected by mutations in genes we aim to test in pharynx muscle, but which may also affect neuronal function, did not influence optogenetic pacing of pharynx muscle.

### Mutations in the EGL-19 L-type voltage gated Ca^2+^channel affect pump duration

**Figure 4:**
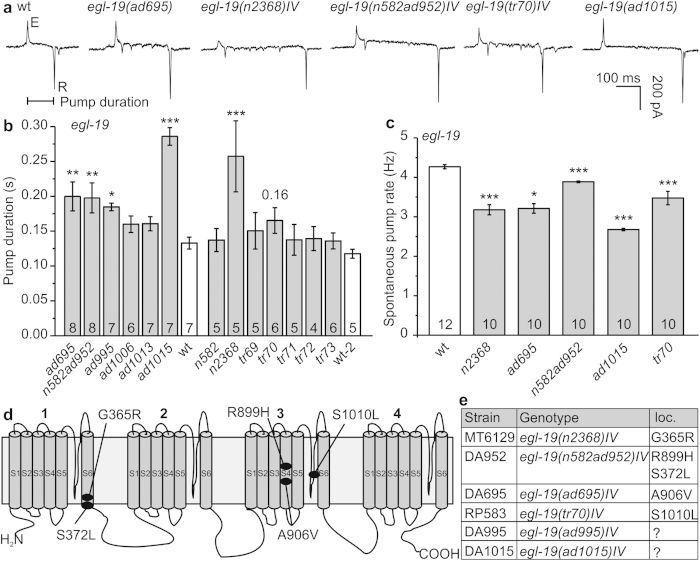
Mutations in the Cav1.2 homolog EGL-19 prolong spontaneous pump duration and reduce pump rate. (**a**) Original EPG recordings of wild type (wt) and *egl-19* mutants (alleles are indicated) with prolonged pump duration (interval E-R spike is indicated). (**b**) Group data, pump durations (mean ± s.e.m.) during spontaneous pumping, of several *egl-19* alleles; statistically significant differences to wt are indicated (***P < 0.001; **P < 0.01; *P < 0.05; one way ANOVA with Fisher post-hoc test). (**c**) Group data, spontaneous pump rate on food of selected *egl-19* mutants, compared to wt (t-test with Bonferroni correction: ***P < 0.001; **P < 0.01; *P < 0.05). (**d**) Schematic of the EGL-19 α1 subunit of the L-type VGCC with the amino acid changes in the alleles listed in (**e**).

We next explored whether mutations in a gene required for function and rhythmicity of the pharynx may be analyzed by optogenetic pacing. L-type VGCCs shape the plateau phase of cardiac, as well as pharyngeal APs. EGL-19 is homologous to human Cav1.2. Numerous alleles of *egl-19* have been isolated, and some of them characterized and classified as reduction- or gain-of-function (r.o.f., g.o.f.) mutants, following different assays (in some cases by electrophysiology), based on EGL-19’s roles in development, pharynx, locomotion and egg-laying muscles^22^,^39^,^40^,^41^,^42^,^43^,^44^. We determined pump durations for a range of these mutants: Alleles *ad695*, *n582ad952*, *ad995*, *ad1015*, and *n2368* showed increased pump duration (Fig. 4a,b), which was accompanied by a significantly reduced pump rate for these alleles, as well as for *tr70* (Fig. 4c). The most pronounced increases of pump duration (~2.1-fold) were found for *ad1015* (counterintuitively, a r.o.f. mutant; another r.o.f. allele, *n582*, did not show altered pump duration) and *n2368* (g.o.f.; slowed inactivation^43^), the latter of which carries the mutation G365R in the S6 transmembrane (TM) segment of the first module (Fig. 4d,e; Supplementary Fig. S6, S7). This mutation affects the equivalent residue as an established LQT8 mutation in Cav1.2, G402S, thus both mutations replace glycine with charged or polar amino acids^11^,^45^. In other g.o.f. alleles, the effects were less pronounced, i.e. ~1.5-fold for *ad695* and *n582ad952*, harboring mutations A906V and the double mutation S372L; R899H^39^, respectively. Allele *tr70* carries the mutation S1010L^42^. All of these residues are within TM segments across modules I or III (R899 and A906 are in the voltage sensor helix S4 of the third module; S1010 is in the pore loop of the third module), or close to the cytosolic membrane face (S372; Fig. 4d,e; Supplementary Figs. S6 and S7, structural model based on the NavAb voltage gated Na^+^ channel^46^). How these mutations may affect EGL-19 desensitization requires detailed analysis; however *ad695* and *n2368* g.o.f. alleles showed reduced desensitization in electrophysiology^43^.

**Figure 5:**
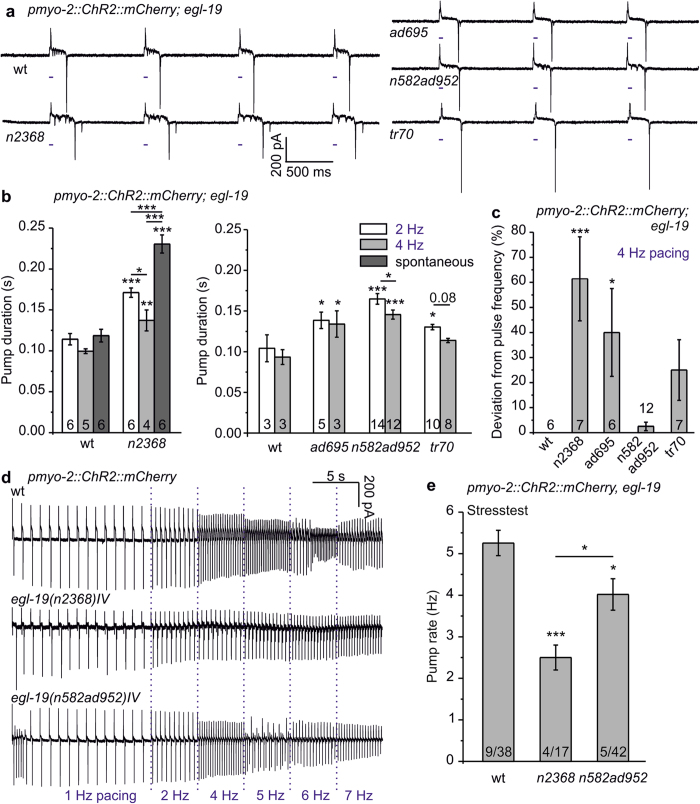
EGL-19 L-type VGCC mutants affect pump parameters and pacing ability of the optically paced pharynx. (**a**) Comparison of EPG recordings of 1 Hz optically paced *egl-19* mutants and wild type (wt). Light pulses are indicated by blue tick marks. (**b**) Group data of pump durations (mean ± s.e.m.; n = 3–10 pharynxes, as indicated in each bar) in pharynxes of *egl-19* alleles, paced at 2 Hz or 4 Hz, as indicated, and compared to wild type (wt) pump durations. Left panel shows recordings of allele *n2368* with 10 ms pacing and exemplary spontaneous pump duration, right panel contains other alleles with 35 ms pacing. (**c**) Deviation of observed pharynx pumping from the pacing frequency, for the indicated fraction of the stimulation period, given in %, for wt and the indicated *egl-19* alleles (n = 6–12 pharynxes, as indicated). (**d**) Maximum pump frequency determined from pacing stress test (optical stimulation: 1 Hz 15s, 2, 4, 5, 6, 7 Hz each 5s) of the indicated wt or *egl-19* mutant pharynxes. (**e**) The mean maximal pump rate achieved in the stress test protocol is shown for wt, as well as the indicated *egl-19* mutants (n = 4–9 experimental days and n = 17–42 pharynxes). Statistically significant differences (***P < 0.001; **P < 0.01; *P < 0.05) were determined by one way ANOVA with Fisher post-hoc test in (**b**,**c**,**e**).

We combined *egl-19* alleles with known molecular lesion with PMC-ChR2 for pacing. Stable pharynx pumping could be induced in all of these strains (Fig. 5a), but the mean pump duration was increased, which was statistically significant for all alleles at 2 Hz pacing (Fig. 5b). Interestingly, mean pump duration in *tr70* was not significantly prolonged at 4 Hz pacing, while *n2368*, *ad695*, and *n582ad952* had increased pump duration also at this pace frequency. Pacing affected pump duration particularly for *egl-19(n2368)*: spontaneous pumps lasted ~1.7-fold longer than during 4 Hz pacing (p ≤ 0.001; Figs 4b and 5b); thus, enforced, regular depolarization may affect EGL-19(G365R) such that it desensitizes faster.

While pacing at 4 Hz, compared to wt, deviations from pacing frequency occurred 61 ± 17% of the time in *n2368*, 40 ± 18% (*ad695*), and 25 ± 12%; (*tr70*; the latter was not significant; Fig. 5c). Deviation from 4 Hz pacing did not correlate with increased pump duration in all cases, i.e. despite increased pump duration for *n582ad952* at 4 Hz, it did not deviate from pace frequency (Fig. 5c). To better understand these different behaviors, we assessed the performance of *n2368* and *n582ad952* in the ‘stress test’. Above distinct frequencies, AP duration and repolarization time should exceed the interstimulus interval, which could lead to cessation of pumping, e.g. through depolarization block, expressing a form of ‘arrhythmia’. Alternatively the pharynx may adapt to the pace rate and reduce the pump duration, or it could skip stimuli and pump at reduced frequency. 15 stimuli at 1 Hz induced steady pumping of the pharynx, then the pacing frequency was increased every 5 s up to 7 Hz (Fig. 5d). Across 4–9 experiments on 17–42 animals, on average, wt reached a maximum pump rate of 5.26 ± 0.3 Hz, whereas *n2368* (2.5 ± 0.3 Hz) and *n582ad952* (4.0 ± 0.3 Hz) mutants showed a reduced ability to pump at higher rates (Fig. 5e). More specifically, 5 Hz was reached by 76.3% of wt, 33.3% of *n582ad952* and only 17.6% of *n2368* animals. 6 Hz pacing was achieved by 44.7% and 11.9% (wt and *n582ad952*, respectively). 7 Hz was achieved by 13.2% of wt, but none of the mutants. Instead, these pharynxes pumped at reduced rate (i.e. only every 2^nd^ or 3^rd^ stimulus evoked a pump; Fig. 5d). Thus, optogenetic pacing facilitates analyzing parameters of rhythmic pharyngeal activity under steady, regular conditions, particularly for mutations affecting pump duration. Several *egl-19* mutations evoked a prolonged ‘Q-T’ (E-R in the pharynx) interval, and at least one allele (*n2368*), resembling the human LQT8 mutation G402S, evoked pharyngeal ‘arrhythmia’ while pacing.

### Achieving high throughput investigation of pumping by video microscopy and kymograph analysis

To further explore using the pharynx as a simple, genetically amenable model for (homologous) mutations affecting human cardiac physiology and arrhythmia, we wanted to analyze drug effects. Conceivably, even drugs specifically addressing distinct molecular lesions may thus be identified, but high throughput would be required, which cannot be easily provided by EPG recordings. Though higher throughput electrophysiological methods, involving microfluidic devices, were described^47^, these are experimentally demanding, problematic with mutant strains growing to small sizes, and not available in many labs. We thus turned to video microscopy, of many animals in parallel, for simple, reliable and versatile analysis of pharynx pumping, enabling access for drugs, and still providing all relevant information about pump rate and duration like EPG recordings.

**Figure 6:**
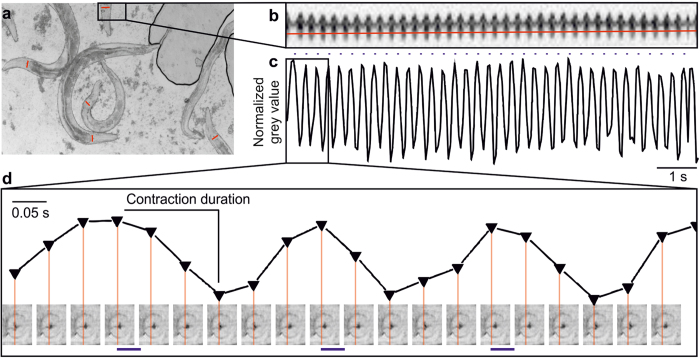
Optical recording of pharynx pumping enabled by kymographic analysis in many animals in parallel. (**a**) Micrograph showing several intact animals, immobilized on polystyrene beads. Red lines drawn across the pharynx terminal bulb indicate lines for which kymographic analysis is performed (grey values along the line obtained from each video frame). (**b**) Kymograph showing grey values of the line depicted in a (a still image of the respective video), upon optogenetic stimulation at 3.7 Hz frequency (35 ms, 470 nm light), for about 30 s. A line scan (red line) across the kymograph is used for detection of pump events, in a plot of grey values as shown in (**c**,**d**) Pump parameters are analyzed with a custom written KNIME script, using maximum and minimum grey values as an indication of axial movement of the pharynx grinder, during a pump cycle. Correlation of grey values with the actual images from the corresponding video frames is shown in the lower half. Red lines represent the line for detection of grinder movement (x-axis—time, y-axis—normalized grey value).

We immobilized intact animals on high-percentage agarose pads with polystyrene beads^48^, and photostimulated them at 3.7 Hz (35 ms, 470 nm; Fig. 6a). Videos were obtained with a high-speed, high-resolution sCMOS camera with large chip size, to film 5–10 animals simultaneously. Pharynxes could easily be visualized at sufficient resolution to quantify pumping, using kymographic analysis. A line drawn across the terminal bulb (Fig. 6a) was used to analyze changes in grey values in each video frame (at 20 fps). Grinder movement could be extracted for periods of 30 s or more, while pumping was photostimulated. Grey values changed synchronously with pumping (Fig. 6b,c) and were quantitatively analyzed using a custom-written KNIME^49^ script. Contraction duration was determined from plotting normalized grey values of kymographic line scans, which could be assigned to single frame images of grinder movement. Onset of grinder displacement corresponds to the start (i.e. the large E-peak in the EPG), and maximal displacement matches the end of the contraction duration period (Fig. 6d; corresponding to the small r-peak in the EPG, which is often not well resolved; thus, generally the E-R duration is extracted from EPGs, see below).

### Arrhythmic pumping behavior of *egl-19* L-type VGCC alleles under pacing conditions

**Figure 7:**
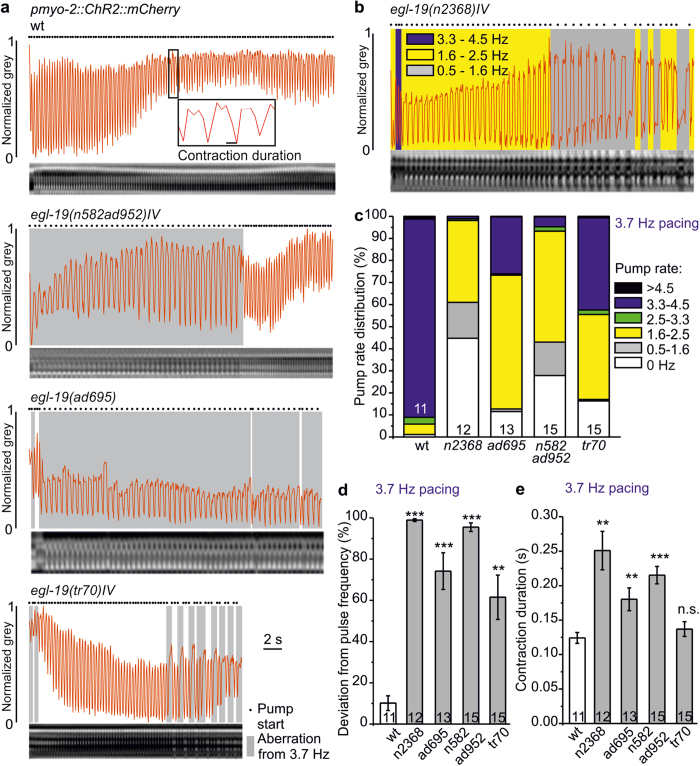
*egl-19* mutants exhibit highly arrhythmic pumping despite steady optical pacing, as shown by kymographic analyses. (**a**) Kymographic analyses of *egl-19* mutant animals compared to wild type (wt), as indicated, during 3.7 Hz pacing (35 ms light pulses, over 30 s). Kymographs are shown below in each panel; the derived normalized grey values are shown as red traces. Periods of observed pumping deviating from 3.7 Hz are shaded grey. Pump starts are shown by black dots. (**b**) Pump rate distribution at 3.7 Hz pacing in a *egl-19(n2368)IV* animal represented by shaded areas (blue: 3.3–4.5 Hz, yellow: 1.6–2.5 Hz, grey: 0.5–1.6 Hz. (**c**) Group data, distributions of pump rates (black: >4.5 Hz, blue: 3.3–4.5 Hz, green: 2.5–3.3 Hz, yellow: 1.6–2.5 Hz, grey: 0.5–1.6 Hz, white: 0 Hz) in the indicated *egl-19* mutants, compared to wild type (wt), obtained from animals paced at 3.7 Hz (n = 11–15 animals). (**d**) Mean deviation (±s.e.m.) of observed pharynx pumping from the pacing frequency, for the indicated fraction of stimulation period, given in % for wild type and the indicated *egl-19* alleles (n = 11–15 animals). (**e**) Contraction duration (mean ± s.e.m.) of the animals analyzed in d. Statistically significant differences in (**d**,**e**): t-test with Bonferroni correction (***P < 0.001; **P < 0.01; *P < 0.05).

We compared pumping parameters obtained from kymographs using 3.7 Hz pacing for wt and *egl-19* alleles *n582ad952*, *ad695*, *tr70* and *n2368* (Fig. 7a,b), to data obtained from EPGs. *egl-19* mutants were often unable to lock in to 3.7 Hz, thus pumping at reduced frequency for variable periods before returning to pace frequency. We statistically analyzed these periods of different pump rates, and the overall deviation from pace frequency (Fig. 7c,d). Resulting pump rate distributions (Fig. 7c) showed that wt pumped at pace frequency ~90% of the time, while *egl-19* mutants showed characteristic distributions of pump frequencies (>4.5 Hz, 4.5–3.3 Hz, 3.3–2.5 Hz, 2.5–1.6 Hz, 1.6–0.5 Hz, and no pumping, 0 Hz), that differed for each of the alleles. This was most obvious for *n2368* (G365R, most similar to the LQT8 mutation G402S^45^), which achieved ~4 Hz pump rate for only ~1% of the time, while it pumped below 2.5 Hz for 98%, and below 1.6 Hz for ~61% of the time. *n582ad952*, *ad695* and *tr70* animals pumped around 4 Hz only 4.6, 25.8 and 41.8% of the time, respectively. Consequently, *egl-19* defective animals pumped at ~2 Hz more often than wt: 37.1% (*n2368*), 60.6% (*ad695*), 50.3% (*n582ad952*) and 38.5% (*tr70*), compared to 4.7% (wt) Overall, all tested *egl-19* mutants deviated significantly from the 3.7 Hz pacing frequency (Fig. 7d). Contraction duration (kymographs, intact wt animals: 124 ± 8 ms; Fig. 7e; EPG recordings, 4 Hz, cut head preparations: 93 ± 9 ms to 100 ± 3 ms, Fig. 5b), was increased in *egl-19* mutants also in kymographs (~2-fold in *n2368*, 1.5-fold in *ad695* and 1.7-fold in *n582ad952*; Fig. 7e). When averaged for periods of similar pump rates (e.g. for ~2 and ~4 Hz; Supplementary Fig. S3), contraction durations of *egl-19* mutants were strongly increased only for 2 Hz pumping, consistent with EPG-derived pump durations. Importantly, altered pump durations and frequency distributions were not due to neuronal effects of *egl-19* mutations, as neuronal photoinhibition in *n2368* mutants neither altered contraction duration nor induced further deviation from pacing frequency (Supplementary Fig. S4).

### Nema-A, a dihydropyridine VGCC antagonist, alters pump duration *egl-19* allele-specifically

**Figure 8:**
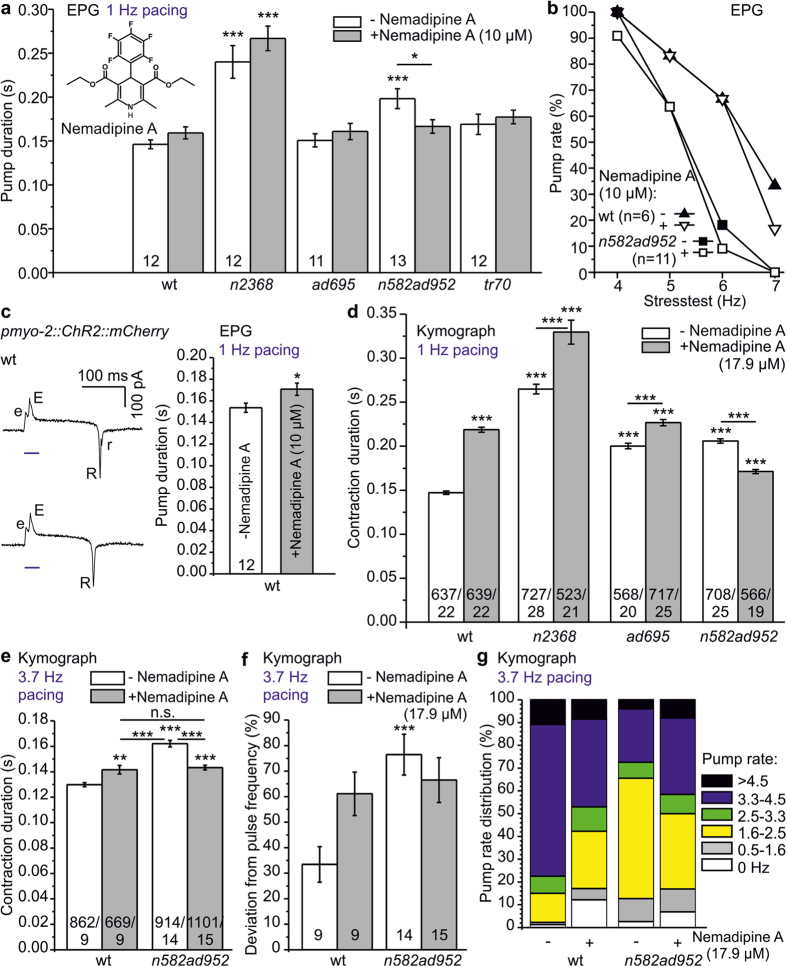
Nema-A ameliorates *egl-19(n582ad952)* mutant defects in paced pumping in dissected pharynxes and intact animals. (**a**) Pump duration deduced from EPG recordings of dissected, optically paced pharynxes (1 Hz), before (white), or following 2 min incubation with nema-A (grey bars, 10 μM; molecular structure shown in the inset), in *egl-19* mutants and wild type (wt) as indicated (n = 11–13 pharynxes). (**b**) Pacing stress test of wt (triangles) and *egl-19(n582ad952)* (squares), following incubation with (10 μM, open symbols) or without nema-A (closed symbols). Shown is the fraction of pharynxes achieving pumping at the indicated pace frequency. (**c**) Not every EPG shows a distinct r-peak. Exemplary analysis of pump duration, as time between E- and r-peak, points out a significant effect of nema-A on wt. (**d**) Contraction duration for the indicated wt or *egl-19* mutant animals, during 1 Hz optical pacing, after 30 min incubation in the absence or presence of 17.9 μM nema-A (n = 19–28 animals, 523–727 contractions). (**e**) Contraction duration, (**f**) mean deviation from pace frequency, and (**g**) pump rate distributions for the indicated wt or *egl-19* mutant animals, during 3.7 Hz optical pacing, after 30 min incubation in the absence or presence of 17.9 μM nema-A, dissolved in M9 buffer, were analyzed by kymographic analysis for higher throughput and detailed pump rate assessment (n = 9–15 animals, 669–1101 contractions). Pump rate distribution in (**g**) is color coded: black: >4.5 Hz, blue: 3.3–4.5 Hz, green: 2.5–3.3 Hz, yellow: 1.6–2.5 Hz, grey: 0.5–1.6 Hz, white: 0 Hz. Statistically significant differences: one way ANOVA with Fisher post-hoc test in (**a**), and t-test with Bonferroni correction in (**c**–**f**) (***P < 0.001; **P < 0.01; *P < 0.05).

Last, we tested optogenetic pharynx pacing for analyzing drugs affecting relevant proteins. Nema-A, a DHP analog (Fig. 8a, inset), is a specific antagonist of *egl-19* in *C. elegans*, but also acts on vertebrate L-type VGCCs^41^,^42^. Nema-A induces various defects in wt growth, morphology and egg-laying, and *egl-19* g.o.f. alleles rescue nema-A induced phenotypes (and vice versa, nema-A rescues some *egl-19* g.o.f. defects)^41^,^42^. Effects on pharynx pumping were not described yet.

Buffer, with or without 0.1% DMSO (used to keep nema-A dissolved), had no effects on pump duration (Supplementary Fig. S5). In EPG analyses at 1 Hz pacing (Fig. 8a), and in a stress test (Fig. 8b), application of nema-A (10 μM, 2 min) to exposed pharynxes in the cut-head preparation, surprisingly, had no significant effect on pump duration of wt or *egl-19* alleles *n2368*, *ad695* and *tr70*. However, as the ‘Auto EPG’ analysis software we used cannot reliably detect small r-peaks in EPGs (i.e. terminal bulb relaxation; Fig. 8c), EPG pump duration is calculated as E-R interval. This is different from kymographic analysis, where onset of terminal bulb contraction and relaxation are measured, corresponding to the E-r interval, which is longer than E-R intervals. To exemplify this for wt, we manually analyzed EPGs with clearly detectable r-peaks, unraveling significantly increased pump duration after nema-A exposure (Fig. 8c). This agreed well with kymographic analyses, where nema-A (17.9 μM for 30 min, as intact animals were used) evoked significantly increased contraction duration by ~33%, ~12% and ~20% in wt, *ad695* and *n2368*, at 1 Hz (Fig. 8d,e), and ~7% for wt at 4 Hz pacing (Fig. 8e). Interestingly, *n582ad952* pump duration was significantly reduced after nema-A treatment in EPG and kymograph analyses at 1 Hz (~16% and ~17%, respectively; Fig. 8a,d) and ~12% at 3.7 Hz (kymograph; Fig. 8e). The ameliorating effect of nema-A on *egl-19(n582ad952)*, a g.o.f. allele combined with a r.o.f. mutation (*n582* had no pumping phenotype on its own; Fig. 4b), occurred in an apparently allele-specific manner: Despite similarly prolonged pump durations in *n2368* and *ad695* g.o.f. animals without nema-A (Figs 4b and 5b), the antagonist further prolonged pumps, just as in wt. Nema-A (10 μM) did not significantly enhance the pump rate of *egl-19(n582ad952)* in the EPG stress test (Fig. 8b), but deviation from pulse frequency, as well as pump rate distribution were reverted to wt (Fig. 8f,g; kymographs, 17.9 μM).

## Discussion

We established the *C. elegans* pharynx as a model for analysis of arrhythmogenic mutations in the EGL-19 L-type VGCC, some of which closely related to human mutations effecting Timothy syndrome. Optogenetic pacing enabled steady pumping up to 7 Hz for short periods, and at 2 or 4 Hz for times >1 min. High reproducibility of stimulation, with ~8 ms between light pulse and terminal bulb contraction (E-peak), as well as low jitter emphasize the quality of the model. For high throughput, we established a video analysis assay of pumping, using kymographic quantification. EPG as well as kymographic analyses demonstrated effects of nema-A, a DHP analog affecting EGL-19 function, in restoring normal pump parameters of one g.o.f. allele.

The optogenetically paced pharynx was not appreciably affected by neuronal input. Three pharyngeal neurons affect normal feeding^15^: M4 is required for isthmus peristalsis^17^, while M3 influences timing of terminal bulb relaxation^50^, and MC may modulate intrinsic pacing of pumping^51^. M4 and MC are cholinergic, and their hyperpolarization (NpHR-mediated), while significantly reducing spontaneous pump rate, had no effect on pump frequency or duration upon PMC-ChR2-pacing. As EGL-19 is expressed also in M4 (ref. 39), we assessed whether *egl-19* mutations may affect paced pumping neuronally. However, while *egl-19(n2368)* pharynxes showed highly aberrant pumping, cholinergic neuron hyperpolarization did not exacerbate their pump phenotypes (Supplementary Fig. S4). Moreover, *unc-13(s69)* animals with abolished chemical synaptic transmission^37^ had unaltered pump duration during pacing.

To probe EGL-19 mutants in modeling LQT8-like arrhythmia in the pharynx, we analyzed several alleles. *ad695*, *n582ad952*, *ad995*, *ad1015*, *n2368* and *tr70* showed increased pump duration and reduced spontaneous pump rate on food. Optogenetic pacing of these alleles uncovered pumping phenotypes resembling LQT8 syndrome: Prolonged pump duration, equivalent to prolonged APs, was likely caused by decreased desensitization of the channel, as established for *n2368* and *ad695* alleles^43^. However, also a r.o.f. allele, *ad1015* prolonged pump duration, for unknown reasons, but possibly pointing to specific roles of EGL-19 in pharynx vs. other muscles.

All of these alleles reduced pump rate, despite the mutated residues being located in different regions of the channel (Supplementary Figs. S6, S7). Gain-of-function alleles *ad695, n582ad952* and *n2368* were previously described to be myotonic and to cause terminal bulb relaxation deficiency; the extent of the effects was *n2368* *>* *ad695* ≫ *n582ad952*^39^, in line with patch clamp measurements for *n2368* and *ad695*^43^. In the context of paced pumping, this translated into arrhythmia. *n2368* showed highest pump duration in spontaneous and paced pumping, and also had the highest deviation from pacing frequency. The *n2368* mutation G365R locates near the cytosolic end of S6 of module I, affecting the equivalent residue of a confirmed human LQT8 mutation (G402S^45^). Given its position in the protein, in structural alignments to the known NavAb structure^46^,^52^ (a surrogate for VGCC structures; Supplementary Figs. S6, S7), this mutation could affect channel desensitization by destabilizing the channel gate. Despite similarly increased pump duration, *n582ad952* did not show aberrant pumping in EPGs, while it did so in kymographic analyses. Possibly, regular, forced pumping induces beneficial effects for this double allele; alternatively, the E-R interval duration is not as well-suited for uncovering arrhythmias as the E-r interval (which became obvious in our nema-A assays). S372L (in *n582ad952*, together with R899H in the S4 helix of the voltage sensor of module III) is very close to G365R. The mutated residue (A906V) in *ad695,* also located in the S4 helix of the voltage sensor of module III, may affect channel gating or repolarization-dependent closure. The S1010L mutation in *tr70*, prolonging pump duration and previously described as mildly myotonic and nema-A resistant (however, with respect to phenotypes other than pharynx pumping)^42^, is located in the pore loop of the third module. Residues in the analogous region of voltage gated K^ + ^channels affect C-type inactivation^53^; however, this type of inactivation has been not reported for VGCCs. Interestingly, also the r.o.f. mutation *ad1015* led to prolonged pump duration, pointing to differences in EGL-19 function in pharynx versus other muscle.

*egl-19* mutations affected the ability of the pharynx to lock-in to fast pacing. In stress tests, using cut head preparations, these mutants could not follow 5–6 Hz pacing, and in intact animals, they often switched from the induced pace rate to lower pump rates, following only every 2^nd^ or 3^rd^ stimulus. Similar effects are observed in LQT8 patients upon increased heart rate in the form of T-wave alternans. Pharynx anatomy is probably too simple to show such effects, yet missed pharynx pumps and T-wave alternans likely have the same origin, i.e. both LQT8 hearts and the pharynx model have prolonged Q-T and E-r interval, respectively. Using the pharynx analysis methods we established, *egl-19* mutants can be recognized as abnormal in paced pumping, and relevant *egl-19* mutations may thus serve as approximated LQT8 models: If novel anti-arrhythmogenic drugs ameliorate aberrant pharynx pumping in such mutants, they may likely act also in LQT8 patients. As we showed, Nema-A reverted prolonged contraction and arrhythmogenic behavior of the *n582ad952* allele in paced pharynxes. In other g.o.f. alleles (*ad695*, *n2368*), nema-A, surprisingly, prolonged pump duration. As nema-A enhances desensitization, it should have effected a r.o.f. phenotype—yet, since the r.o.f. allele *ad1015* also prolonged pump duration, this may be a specific consequence of EGL-19 r.o.f. in (paced) pharynx pumping. Our findings indicate allele-specific drug effects of nema-A, at least in our assay. Thus, patient-specific mutations may be introduced into the pharyngeal EGL-19 channel, and patient-specific drugs could be identified from drug libraries devised for Cav1.2.

In sum, we established an optogenetic pacing system in the pharynx of the genetically amenable animal model *C. elegans*, enabling to (introduce and) study mutations in genes required for normal pharynx pumping and rhythmicity, including orthologous human cardiac disease associated arrhythmogenic mutations. Our approach enables drug-testing and, using kymograph analysis, even to screen drug libraries. It should be transferable to other arrhythmia types with genetic causes, e.g. catecholaminergic polymorphic ventricular tachycardia (CPVT)^10^.

## Methods

### *C. elegans* strains

*C. elegans* strains were cultivated at 20 °C either on nematode growth medium (NGM)^54^ or for electrophysiological experiments on high growth medium (HGM), fed with *E. coli* strain OP-50-1. We used the following strains: N2 (wild type), **DA695:** *egl-19(ad695)*, **DA952:** *egl-19(n582ad952)*, **DA995:** *egl-19(ad995)*, **DA1006:** *egl-19(ad1006)*, **DA1013:** *egl-19(ad1013)*, **DA1015:** *egl-19(ad1015)*, **MT1212:** *egl-19(n582)*, **MT6129:** *egl-19(n2368)*, **RP582:** *egl-19(tr69)*, **RP583:** *egl-19(tr70)*, **RP584:** *egl-19(tr71)*, **RP585:** *egl-19(tr72)*, **RP586:** *egl-19(tr73)*. *unc-13(s69)* was kindly provided by E. Jorgensen.

We generated these transgenic strains: **ZX1652:** *egl-19(n2368); zxIs20[pmyo-2::ChR2(H134R)::mCherry; pges-1::nls::GFP]*, **ZX1661:** *egl-19(n582ad952); zxIs20[pmyo-2::ChR2(H134R)::mCherry; pges-1::nls::GFP]*, **ZX1662:** *N2; zxIs20[pmyo-2::ChR2(H134R)::mCherry; pges-1::nls::GFP]*, **ZX1808:** *egl-19(ad695); zxIs20[pmyo-2::ChR2(H134R)::mCherry; pges-1::nls::GFP]*, **ZX1809:** *egl-19(tr70); zxIs20[pmyo-2::ChR2(H134R)::mCherry; pges-1::nls::GFP]*, **ZX1810:** *unc-13(s69); zxIs20[pmyo-2::ChR2(H134R)::mCherry; pges-1::nls::GFP]*, **ZX1811:** *zxEx796[punc-17::NpHR::YFP;pelt-2::mCherry]; zxIs20[pmyo-2::ChR2(H134R)::mCherry; pges-1::nls::GFP],* **ZX1812:** *egl-19(n2368); zxEx797[punc-17::NpHR::YFP;pelt-2::mCherry]; zxIs20[pmyo-2::ChR2(H134R)::mCherry; pges-1::nls::GFP]*

### Plasmids

Plasmid pmyo-2::ChR2(H134R)::mCherry was generated by PCR amplification of the *myo-2* promotor (primers **oEF3**: 5′-ACATGTCAGGTCGAGGCATTTG-3′ and **oEF4**: 5′-GGATCCCCCGAGGGTTAAAATGA-3′) from pPD132.102 (Addgene). The amplicon was digested with BamHI and PciI and ligated into plasmid pdat-1::ChR2::mCherry^55^. Punc-17::NpHR::YFP was described previously^38^.

### Microinjection and generation of transgenic animals

Animals expressing ChR2 in PMCs were generated by microinjection into the germline (3 ng/μL pmyo-2::ChR2::mCherry, 20 ng/μL ges-1::nls::gfp, and 100 ng/μL pUC-19 plasmid). For chromosomal integration, 100 transgenic L4 larvae were irradiated with 66.6 mJ UV light. After starving, F2 generation was washed off with M9 buffer, 1000 transgenic animals were singled and integration events were identified by screening the F3 generation^56^. The integrated transgene *zxIs20* was outcrossed 4x, and then crossed into the candidate *egl-19* mutant strains.

### Genotyping of *egl-19* mutants

Genotyping of *egl-19(n2386)* occurred by phenotype, and was confirmed by sequencing of PCR products (primers **oEF100:** 5′-TCGAGCCATGATTCCTTTGC-3′, **oEF101:** 5′-TCTAGCTGCCCATTTACTCG-3′). *egl-19(ad695)* was genotyped after PCR (**oEF102:** 5′-AGCTGCTGAAGATCCTCTAC-3′ and **oEF103:** 5′-TGGCCATCCTTCGAAAGTTG-3′), the PCR product was digested with HpyCH4V and analyzed by 2% polyacrylamide gel electrophoresis. *egl-19(n582ad952)* was genotyped by MseI digestion of the oEF100/oEF101 amplicon. Genotyping of *egl-19(tr70)* was by differential annealing temperature binding of primers carrying mutated or wt bases at the 3‘-end. Primers for the mutation were **oEF104:** 5′-GCGATGATTTCACTTTTCGTAGTTCT-3′; **oEF106:** 5′-TGCTTGCACGCCAAAGATAC-3′, and **oEF105:** 5′-GCGATGATTTCACTTTTCGTAGTTTC-3′; **oEF106:** 5′-TGCTTGCACGCCAAAGATAC-3′ for wt. Last, all alleles were verified by sequencing.

### Fluorescence microscopy

Expression of pmyo-2::ChR2::mCherry was analyzed on a Zeiss Axio Observer, with an 40x/0.25 Zeiss ∞/- APlan oil objective and mCherry filter set (Ex 580/23 nm, BS 605, Em 625/15 nm). Animals were transferred on 2% agarose pads in M9 buffer (K_2_PO_4,_ 20 mM; Na_2_HPO_4_, 40 mM; NaCl, 80 mM ; MgSO_4,_ 1 mM) and immobilized with 1 μL freshly prepared 50 mM NaN_3_ solution (Sigma-Aldrich, USA, St. Louis) in M9 from a 1 M stock in water.

### Determination of spontaneous pump rate on food

One day before experiments, L4 larvae were placed on NGM dishes (55 mm, 8 mL NGM) seeded with 320 μL OP50 culture. For measurements requiring ATR (Sigma-Aldrich) 1:1000 of a 100 mM stock in ethanol was added to OP50. Pumping of animals on food was visually counted for 1 min. Measurements of animals co-expressing pmyo-2::ChR-2::mCherry and punc-17::NpHR::YFP (strains ZX1811 and ZX1812) were performed on a fluorescence stereo microscope (MZ16F; Leica, Germany) with continuous illumination (590 nm, 1 mW/mm^2^). Mean values, s.e.m. and further statistics (t-test and Bonferroni correction) were calculated with Excel (Microsoft, USA).

### EPG-Recording and Optical Pacing

About 16–24 hours prior to experiments, young adult hermaphrodites were placed on fresh HGM plates with or without ATR. ATR (0.65 μl of a 100 mM stock in ethanol) was added to 650 μl of OP50 culture and spread onto 94 mm culture dishes (vented, Greiner Bio-One) containing 25 mL of HGM. For cut head preparations, animals were transferred into a recording chamber containing a Sylgard-coated coverslip (Ø25 mm) and filled with 1.5 mL of EmD50 buffer (NaCl, 140 mM; KCl, 3 mM; CaCl_2_, 3 mM; MgCl_2_, 1 mM; Hepes, 10 mM; D-Mannitol, 50 mM; pH 7.3 adjusted with NaOH). Dissection was performed under a stereomicroscope (Stemi 2000-C, Zeiss). The head was cut away from the body with a scalpel (Braun Aesculap, Germany) directly posterior to the terminal bulb. Upon dissection, the body wall muscles contract and expose the posterior pharynx^57^.

Electrophysiology was performed on an Olympus BX51W microscope. After positioning the recording chamber, the tip of the worm head was sucked under 100-fold magnification into an EPG-suction electrode, connected via a silver-chloride coated silver wire to a EPC-10 amplifier (Heka, Germany). The EPG-suction electrode was pulled from Borosilicate glass 1B100-4 (World Precision Instruments) by using a horizontal puller (Sutter P97) to ~20 μm inner diameter tip. As a bath electrode an Ag/AgCl pellet electrode (World Precision Instruments) was used^27^,^28^. For optical pacing with a 470 nm LED (KSL-70, Rapp Optoelectronics, Germany) the pharynx was positioned below a 60x water-immersion objective (LUMIplan FI/IR, 0.9 NA); an EGFP-ET filter set (AHF Analysentechnik AG, Germany) was used. EPG recording and triggering of light pulses was synchronized by PatchMaster software (Heka).

We recorded spontaneous pumping (at least for 1 minute) or stimulated the pharynx with 470 nm light pulses. 1.5–2 mW/mm^2^ intensity and pulse duration of 10 or 35 ms were most suitable. In every paced experiment, the first five (1 Hz stimulation) or ten pumps (>1 Hz stimulation) were excluded from analysis, to obtain stable pumping. For drug tests, 10 s of 1 Hz stimulation were recorded before and two minutes after drug application. Nema-A (Sigma-Aldrich) stock was prepared in DMSO before dilution in EmD50. 500 μl nema-A (30 μM in EmD50) was applied to the recording chamber containing 1 mL EmD50, resulting in a concentration of 10 μM. Final DMSO concentration was ~0.1%.

We used Review software (Bruxton Corporation, USA) to translate PatchMaster files to ABF files. Pump rate and duration were analyzed by AutoEPG^58^ (kindly provided by C. James, Southampton, UK). Excel was used for calculation of means and s.e.m., 1-way ANOVA with Fisher post-hoc test was performed using OriginPro (OriginLab, USA).

### High-throughput kymograph recording and optical pacing

Transgenic L4 larvae cultivated with ATR were placed on fresh NGM dishes, seeded with OP-50-1 and ATR, one day prior to the assay. 1 μL of polystyrene microspheres (Polysciences 00876–15, 2.5% w/v suspension) were added on pads composed of 10% agarose^59^. About 10 animals were transferred into the beads and gently overlaid with a coverslip^48^.

Measurements were performed on a Zeiss Axio Observer, equipped with a 100 W HBO lamp, EGFP Filter (Ex. 472/30, beam splitter 570, Em. 675/50) and an 10x/0.25 Zeiss ∞/- APlan objective. Animals were stimulated with blue light pulses (35 ms, 1.5 mW/mm^2^) with a computer-controlled shutter (Sutter Instruments) every 250 ms (~4 Hz) or second (~1 Hz) over a 30 s period. Recording was via an ORCA Flash 4.0 sCMOS camera (Hamamatsu, Japan; 20 fps, 10 ms exposure time, 2 × 2 binning, 1024 × 1024 Pixel) and μManager v1.4 software. For drug tests, animals were incubated 30 min in 10 μL 17.9 μM nema-A solution (Sigma-Aldrich) with 0.3% DMSO in M9 (Stock solution 47.7 mM in DMSO). To determine influence of cholinergic neurons on pacing, measurements were performed during continuous illumination (0.5 mW/mm^2^) with a 590 nm LED (KSL-054, Rapp Optoelectronics) by 3.7 Hz stimulation, 35 ms blue light pulses, over 30 seconds.

Multi-kymographs of grinder movements from each animal were prepared using ImageJ. The region of interest was chosen next to the grinder, such that pump movements showed as bright or dark parts in the kymograph. After setting a line scan through the kymograph, pump movements were extracted as periodic grey value changes. Traces were normalized and if necessary inverted to achieve a consistent representation of pump movements, by a custom KNIME script^49^. Start points and end of minima were defined in each trace using manual correction and a machine learning process based on fixed rules. Calculation was restricted to the first 100 light pulses for measurements with stimulation frequencies of ~4 Hz. Pumping frequency was calculated from the beginning of consecutive peaks. Time dependent aberration from 4 Hz was determined if pump rate was >3.3 Hz and <4.5 Hz (±1 frame). Pumping frequencies were classified in groups: 0 Hz, 0.5–1.6 Hz, 1.6–2.5 Hz, 2.5–3.3 Hz, 3.3–4.5 Hz and >4.5 Hz. Contraction duration was calculated from fractions of time between beginning of peak and the end of the subsequent minima in Excel (Microsoft). Furthermore, data was correlated to particular ranges of pumping frequencies to assess correlation between observed pumping frequency and contraction duration. Statistically significant differences were determined by t-test and Bonferroni correction.

### Structural model of EGL-19

The x-ray structure of the homo-tetrameric voltage gated sodium channel NavAb from *Arcobacter butzleri* (PDB file 3RVZ) was used as 3D model for positioning of mutated residues in the *egl-19* alleles tested in this work. Each module of EGL-19 (a monomer) was aligned with NavAb (Supplementary Fig. S7, showing only the first 3 modules), and the likely positions of the residues determined and highlighted in the NavAb structure, rendered in different orientations, using PyMOL (www.pymol.org; Supplementary Fig. S6). Some views were given as 30 Å slabs for better visualization.

## Additional Information

**How to cite this article**: Schüler, C. *et al.* Arrhythmogenic effects of mutated L-type Ca2+-channels on an optogenetically paced muscular pump in *Caenorhabditis elegans*. *Sci. Rep.* **5**, 14427; 10.1038/srep14427 (2015).

## Supplementary Material

Supplementary Information

Supplementary Video 1
