# Supplementary material for: Arrhythmogenic effects of mutated L-type Ca2+-channels on an optogenetically paced muscular pump in Caenorhabditis elegans
Source: Sci Rep. 2015 Sep 24;5:14427. doi: 10.1038/srep14427 (PMC4585839; doi:10.1038/srep14427)
Supplement: Supplementary Information [file srep14427-s1.pdf]

Arrhythmogenic effects of mutated L-type Ca^{2+}-channels on an optogenetically paced muscular pump in *Caenorhabditis elegans*

Christina Schüler[1,2,3*], Elisabeth Fischer[1,2,3,*], Lior Shaltiel[1,2,4], Wagner Steuer Costa[1,2] & Alexander Gottschalk[1,2,3,5]

[1] Buchmann Institute for Molecular Life Sciences, Goethe University, Max von Laue Strasse 15, D-60438 Frankfurt, Germany

[2] Institute of Biochemistry, Goethe University, Max von Laue Strasse 9, D-60438 Frankfurt, Germany

[3] Cluster of Excellence Frankfurt – Macromolecular Complexes, Goethe University, Max von Laue Strasse 15, D-60438 Frankfurt, Germany

[4] Present address: LipoCure Ltd, Hadassah Ein Kerem Campus, Minrav Building, Jerusalem Biotechnology Park, POB 12235, Jerusalem, 9112102 Israel

* these authors contributed equally

[5] to whom correspondence should be addressed: a.gottschalk@em.uni-frankfurt.de

Supplementary Information

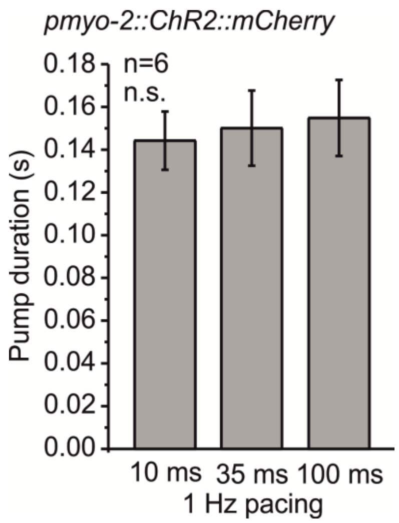

Supplementary Fig. S1: Pump duration is not altered by length of stimulation light pulse (10 - 100 ms)

Pacing with 10, 35 and 100 ms (470 nm) has no effect on pump duration as deduced from EPGs.

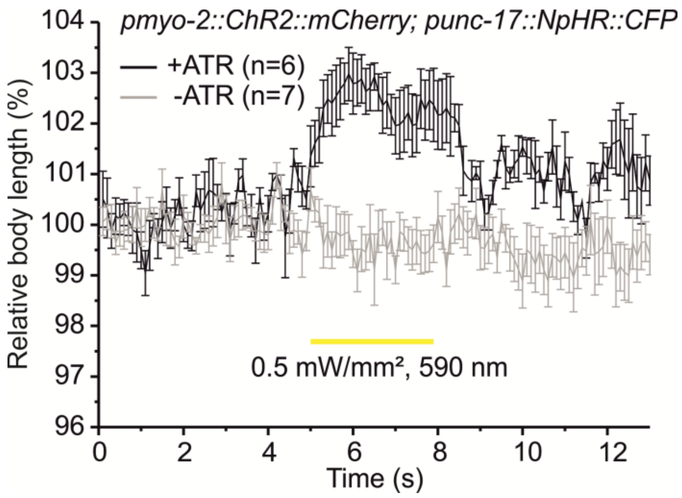

Supplementary Fig. S2: Cholinergic neuron hyperpolarization induces body relaxation

Animals expressing ChR2 in PMCs and NpHR (halorhodopsin) in cholinergic neurons were illuminated with 570-610 nm light (0.5 mW/mm²) for 3 s. Analysis of averaged (± S.E.M.), normalized body length deduced from videos (10 fps) verifies that cholinergic neuron hyperpolarization is effective and induces muscle relaxation (black trace, n=6 animals treated with ATR). No effects are observed in animals grown the absence of ATR (grey trace, n=7 animals). Illumination period is indicated by a yellow bar.

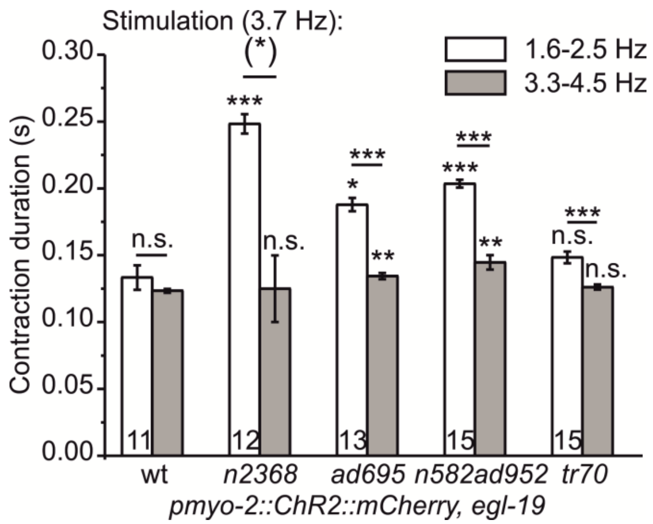

Supplementary Fig. S3: Detailed analysis of contraction duration obtained by kymographic analysis

Detailed analysis shows strongly increased contraction durations only for the 2 Hz pumping periods of *egl-19* mutants compared to wild type (wt). Statistically significant differences: t-test with Bonferroni correction (***P<0.001; **P< 0.01).

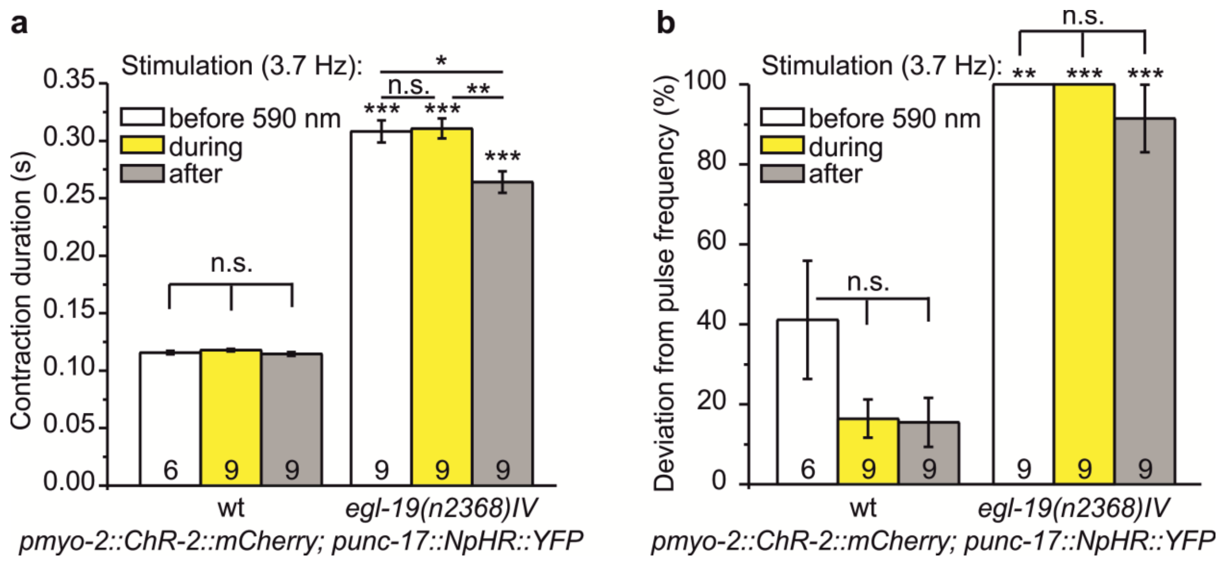

Supplementary Fig. S4: No neuronal effect on contraction duration and deviation from 3.7 Hz pumping of *egl-19* mutants

A) Contraction duration as measured by kymographs upon ongoing ChR2 PMC photostimulation at 3.7 Hz, combined with photoinhibition of cholinergic neurons by NpHR (yellow), in *egl-19(n2368)* mutants, compared to wild type (wt). **B**) Fraction of time the animals in A) showed deviation from the pace frequency before or after cholinergic neuron photoinhibition. Statistically significant differences: t-test with Bonferroni correction (***P<0.001; **P< 0.01; *P<0.05).

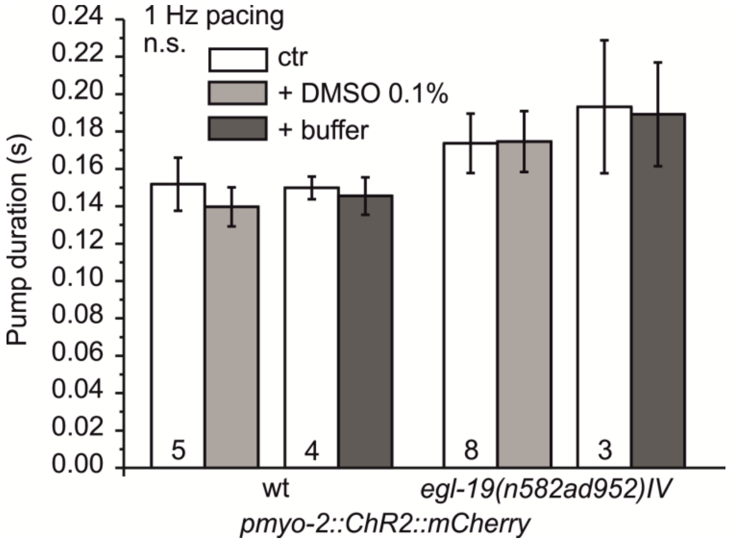

Supplementary Fig. S5: No effect of 0.1% DMSO on pump duration

Pump duration was analyzed by EPGs of 1 Hz optically paced pharynxes, of allele *n582ad952* and compared to wild type (wt), in control conditions, or 2 min after incubation with 0.1% DMSO, or after application of buffer (EmD50).

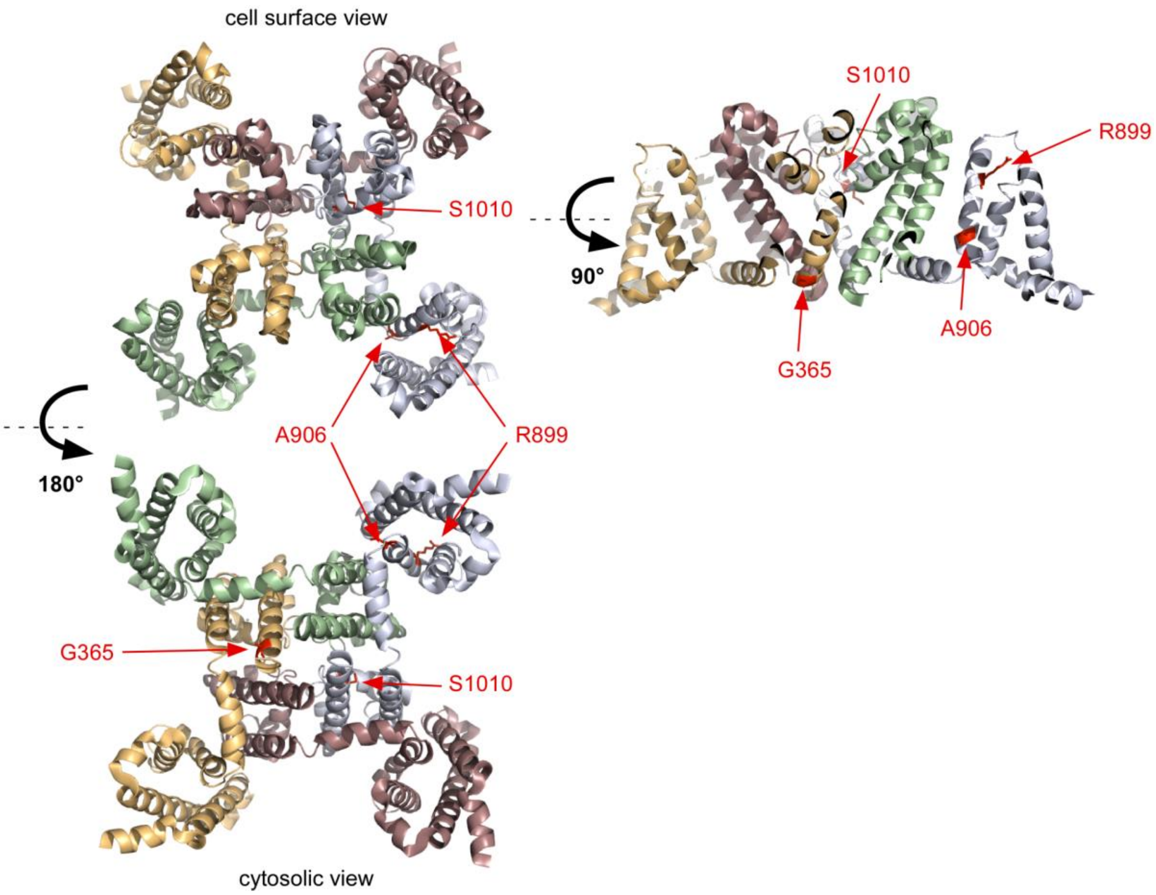

Supplementary Fig. S6: **Structure model of NavAb as EGL-19 surrogate with positions of the analyzed *egl-19* mutations indicated**

Structural model of NavAb (PDB file 3RVZ), as a surrogate for the EGL-19 structure, with relevant, mutated amino acids superimposed (red) over positions of NavAb that align with the EGL-19 sequence (see **Supplementary Fig. S7**).

1st module

```
EGL-19            50 -aqrkplrqtnvversersllclslnnpirklcisivewkpfeflilfmicancialaiyqpypaqdsdy
Cav1.2 human     100 ---------------pprallcltlknpirracisivewkpfeiiilltifancvalaiyipfpeddsna
navAB (3RVZ)       1 mdykddddkgslvprgshmylrit----------nivessfftkfiiylivlngitmgletsktfmqsfg

EGL-19           119 kntlletieyvfivvftiecvlkivamgfmfhpsaylrnawnildfiivviglvstilskmsiq------
Cav1.2 human     155 tnsnlerveylfliiftveaflkviaygllfhpnaylrngwnlldfiivvvglfsaileqatkadganal
navAB (3RVZ)      61 vyttl--fnqivitiftieiilriyv-----hrisffkdpwslfdffvvaislvpt------ss------

EGL-19           183 -----gfdvkalrafrvlrplrlvsgvpslqvvlnailramipllhiallvlfviliyaiiglelfcgkl
Cav1.2 human     225 ggkgagfdvkalrafrvlrplrlvsgvpslqvvlnsiikamvpllhiallvlfviiiyaiiglelfmgkm
navAB (3RVZ)     112 -----gfei--lrvlrvlrlfrlvtavpqmrkivsalisvipgmlsvialmtlffyifaimatqlf----

EGL-19           248 hstcidpa--tgqlaqkdptpcgtdtegsafkcqpsdsltnmgvrwecssnttwpgpnngitnfdnfgla
Cav1.2 human     295 hktcynqegiadvpaeddpspcalet-ghgrqcq-ngtvckpg----------wdgpkhgitnfdnfafa
navAB (3RVZ)     171 ---------------------------gerfp------------ew-----------------fgtlges

EGL-19           316 mltvfqcvslegwtdvmywvndavg-rewpwiyfvtlvilgsffvlnlvlgvlsgefskerekararglf
Cav1.2 human     353 mltvfqcitmegwtdvlywvndavg-rdwpwiyfvtliiigsffvlnlvlgvlsgefskerekakargdf
navAB (3RVZ)     185 fytlfqvmtlesws--mgivrplmevypyawvffipfifvvtfvminlvvaicvdamailnqkeeqhiid
                                                                          G365    S372

EGL-19           385 q-------------kfre-----kqqleedlkgy-------------
Cav1.2 human     422 q-------------klre-----kqqleedlkgyldwitqaedidpe
navAB (3RVZ)     253 evqshedninneiiklreeivelkeliktslkn--------------
```

2nd module

```
EGL-19           440 -vedvrpskwaarmk-rlekl---------nrrcrracrrlvksqtfywlvillvllntlvl---tsehy
Cav1.2 human     480 aggdiegencgarlahriskskfsrywrrwnrfcrrkcraavksnvfywlviflvflntlti---asehy
navAB (3RVZ)       1 -mdykddddkgslvp-rgshm---------ylr----itnivessfftkfiiylivlngitmgletsktf

EGL-19           496 gqsewldhfqtmanlffvilfsmemllkmyslgfttyttsqfnrfdcfvvissilefvlvyfdlmkplgv
Cav1.2 human     547 nqpnwltevqdtankallalftaemllkmyslglqayfvslfnrfdcfvvcggiletilvetkimsplgi
navAB (3RVZ)      56 mqs--fgvyttlfnqivitiftieiilriy-vhrisffkdpwslfdffvvaislvp---------tssgf

EGL-19           566 svlrsarllrifkvtkywtslrnlvssllnslrsiislllllflfivifallgmqvfggkfnfnpqqpkp
Cav1.2 human     617 svlrcvrllrifkitrywnslsnlvasllnsvrsiaslllllflfiiifsllgmqlfggkfnfdemqtr-
navAB (3RVZ)     114 eilrvlrvlrlfrlvtavpqmrkivsalisvipgmlsvialmtlffyifaimatqlfgerf---pew---

EGL-19           636 ranfdtfvqalltvfqiltgedwntvmyhgiesfggvgtlgvi---vciyyivlficgnyillnvflaia
Cav1.2 human     686 rstfdnfpqslltvfqiltgedwnsvmydgimayggpsfpgml---vciyfiilficgnyillnvflaia
navAB (3RVZ)     178 ---fgtlgesfytlfqvmtlesw---------smgivrplmevypyawvffipfifvvtfvminlvvaic

EGL-19           703 vdnladadsltnaekeeeqqeiegedeefee-gedegeehgmdep---egdeemtsarprrm
Cav1.2 human     753 vdnladaesltsaqkeeee---ekerkklartaspekkqelvekpavgeskeekielksit-
navAB (3RVZ)     236 vdama----iln---qkeeqhiidevqshed-ninneiiklreei---velkeliktslkn-
```

3rd module

```
EGL-19           750 --eemtsarprrmsevpaastvkpipkasslfilshtnsfrvfcnmvvnhsyftnavlfcilvssamlaa
Cav1.2 human     850 epempvgprprplselhlkekavpmpeasaffifssnnrfrlqchrivndtiftnlilffillssislaa
navAB (3RVZ)       1 ------------mdykddddkgslvprgshmy-lritn--------ivessfftkfiiylivlngitmgl

EGL-19           818 edplqanstrnmilnyfd--------------------yfftsvftveitlkvivfglvfhkgsfcrnaf
Cav1.2 human     920 edpvqhtsfrnhilfyfdivfttiftieialkilgnadyvftsiftleiilkmtaygaflhkgsfcrnyf
navAB (3RVZ)      50 etsktfmqsfgvyttlfn--------------------qivitiftieiilriyv-----hrisffkdpw

EGL-19           868 nlldilvvavsltsfvlrtdamsvvkilrvlrvlrplrainrakglkhvvqcvivavktignimlvtfml
Cav1.2 human     990 nildllvvsvslisfgiqssainvvkilrvlrvlrplrainrakglkhvvqcvfvairtignivivttll
navAB (3RVZ)      95 slfdffvvaisl------vptssgfeilrvlrvlrlfrlvtavpqmrkivsalisvipgmlsvialmtlf
                                                    R899    A906

EGL-19           938 qfmfaiigvqlfkgtfflcndlskmteaecrgeyihyedgdptkpvskkrvwsnndfnfdnvgdamislf
Cav1.2 human    1060 qfmfacigvqlfkgklytcsdsskqteaeckgnyitykdgevdhpiiqprswenskfdfdnvlaammalf
navAB (3RVZ)     159 fyifaimatqlfgerf---------------------------------pew------fgtlgesfytlf

EGL-19          1008 vvstfegwpqllyvaidsneedkgpihnsrqavalffiafiiviaffmmnifvgfvivtfqnegereyen
Cav1.2 human    1130 tvstfegwpellyrsidshtedkgpiynyrveisiffiiyiiiiaffmmnifvgfvivtfqeqgeqeykn
navAB (3RVZ)     190 qvmtleswsmgiv----------rplmevypyawvffipfifvvtfvminlvvaicvdamailnqkeeqh
                     S1010

EGL-19          1078 celdknqrkciefalkakphrryiprnrlqyrvwwfvtsrafeyvifliivmntvslackhypssrgfed
Cav1.2 human    1200 celdknqrqcveyalkarplrryipknqhqykvwyvvnstyfeylmfvlil-------------------
navAB (3RVZ)     250 i-idevq-----------shedninneiiklr----------eeivelkelikt---slkn---------
```

Supplementary Fig. S7: **Sequence alignments of NavAb with each of the first 3 modules of EGL-19**

Supplementary Video 1: Pharynx in electropharyngeogram setup; first spontaneous pumps, then optically paced at 1, 2 and 4 Hz
